# Activity of fosfomycin against nosocomial multiresistant bacterial pathogens from Croatia: a multicentric study

**DOI:** 10.3325/cmj.2018.59.56

**Published:** 2018-04

**Authors:** Luka Bielen, Robert Likić, Viktorija Erdeljić, Ivana Mareković, Nataša Firis, Marijana Grgić-Medić, Ana Godan, Ivan Tomić, Blaženka Hunjak, Alemka Markotić, Danijela Bejuk, Vladimira Tičić, Silvana Balzar, Branka Bedenić

**Affiliations:** 1 Division of Intensive Care, Department of Internal Medicine, University Hospital Center Zagreb, Zagreb, Croatia; 2 University of Zagreb School of Medicine, Zagreb, Croatia; 3 Division of Clinical Pharmacology, Department of Internal Medicine, University Hospital Center Zagreb, Zagreb, Croatia; 4 Clinical Department of Clinical and Molecular Microbiology, University Hospital Center Zagreb, Zagreb, Croatia; 5 Croatian National Institute of Public Health, Zagreb, Croatia; 6 Clinic for Infectious Diseases “Dr. Fran Mihaljević,” Zagreb, Croatia; 7 Clinical Hospital “Sveti Duh,” Zagreb, Croatia; 8 “Andrija Štampar” Teaching Institute of Public Health, Zagreb, Croatia; 9 Polyclinic Breyer for Medical Biochemistry and Microbiology, Zagreb, Croatia

## Abstract

**Aim:**

To determine *in vitro* susceptibility of multiresistant bacterial isolates to fosfomycin.

**Methods:**

In this prospective *in vitro* study (local non-random sample, level of evidence 3), 288 consecutively collected multiresistant bacterial isolates from seven medical centers in Croatia were tested from February 2014 until October 2016 for susceptibility to fosfomycin and other antibiotics according to Clinical and Laboratory Standards Institute methodology. Susceptibility to fosfomycin was determined by agar dilution method, while disc diffusion were performed for *in vitro* testing of other antibiotics. Polymerase chain reaction and sequencing was performed for the majority of extended spectrum β-lactamase (ESBL)-producing *Klebsiella pneumoniae* (*K. pneumoniae*) and carbapenem-resistant isolates.

**Results:**

The majority of 288 multiresistant bacterial isolates (82.6%) were susceptible to fosfomycin. The 236 multiresistant Gram-negative isolates showed excellent susceptibility to fosfomycin. Susceptibility rates were as follows: *Escherichia coli* ESBL 97%, *K. pneumoniae* ESBL 80%, *Enterobacter species* 85.7%, *Citrobacter freundii* 100%, *Proteus mirabilis* 93%, and *Pseudomonas aeruginosa* 60%. Of the 52 multiresistant Gram-positive isolates, methicillin-resistant *Staphylococcus aureus* showed excellent susceptibility to fosfomycin (94.4%) and vancomycin-resistant *enterococcus* showed low susceptibility to fosfomycin (31%). Polymerase chain reaction analysis of 36/50 ESBL-producing *K. pneumoniae* isolates showed that majority of isolates had CTX-M-15 beta lactamase (27/36) preceded by IS*Ecp* insertion sequence. All carbapenem-resistant *Enterobacter* and *Citrobacter* isolates had *bla*_VIM-1_ metallo-beta-lactamase gene.

**Conclusion:**

With the best *in vitro* activity among the tested antibiotics, fosfomycin could be an effective treatment option for infections caused by multiresistant Gram-negative and Gram-positive bacterial strains in the hospital setting.

Fosfomycin is a phosphonic acid derivative with a broad-spectrum antibacterial activity. It inhibits peptidoglycan assembly, thereby disrupting bacterial cell wall synthesis (1). It has been clinically available for decades and a single dose of fosfomycin has been widely accepted as the first line treatment for uncomplicated urinary tract infections (UTIs) (2). In the form of its trometamol salt, approximately 40% of the drug is absorbed following oral administration. After being released from trometamol by hydrolysis, fosfomycin is rapidly excreted unchanged by glomerular filtration and reaches high-peak urinary concentration of approximately 4000 mg/L. Fosfomycin treatment achieves comparable clinical and microbiological cure rates to longer courses of antibiotic alternatives (quinolones, beta lactams, aminoglycosides, nitrofurantoin, and sulfonamides) (3). Moreover, a number of *in vitro* studies have demonstrated that fosfomycin has an excellent activity against many multiresistant bacteria, including extended spectrum β-lactamase (ESBL) and plasmid-mediated ampicillin class C (AmpC)-producing Gram-negative bacilli and carbapenem-resistant *Enterobacteriaceae* (4,5). Based on the results of previous *in vitro* studies, we hypothesized that the majority of multiresistant Gram-negative and Gram-positive bacteria, including carbapenem-resistant isolates, would exhibit susceptibility to fosfomycin. Compared to the previous studies, we included a larger proportion of carbapenem-resistant isolates, which present a considerable challenge for clinicians. Since the literature data about multiresistant *Citrobacter freundii* (*C. freundii*) susceptibility to fosfomycin is scarce, we also tested a relatively large number of *C. freundii* isolates. Therefore, we tested fosfomycin’s *in vitro* activity against multidrug resistant pathogens for which very limited antibiotic options are available.

## Materials and methods

In this prospective *in vitro* study, with the level of evidence 3, 288 multiresistant bacterial clinical isolates were tested from February 2014 until October 2016 in the Clinical Hospital Center Zagreb for *in vitro* susceptibility to fosfomycin and other antibiotics. Multiresistance was defined as resistance to at least three members of different antibiotic groups (6). Isolates were collected from six medical centers in Zagreb and one in Split (Clinical Hospital Center Zagreb, Croatian National Institute of Public Health, Clinic for Infectious Diseases “Dr. Fran Mihaljević,” Clinical Hospital “Sveti Duh,” “Andrija Štampar” Teaching Institute of Public Health, Polyclinic Breyer for Medical Biochemistry and Microbiology, Clinical Hospital Center Split). The majority of isolates (75%) originated from the University Hospital Center Zagreb. Non-copy clinical isolates were consecutively collected during the period from February 2014 until October 2016. While the vast majority of Gram-negative isolates were from urine, Gram-positive isolates were also collected from other clinical specimens such as blood cultures, stool, ascites, central venous catheters, tracheostomy, throat, and wound swabs. The following number of isolates were tested, all having multiresistant phenotype: *Escherichia coli* (*E. coli*) (n = 72), *Klebsiella pneumoniae* (*K. pneumoniae*) (n = 62), methicillin-resistant *Staphylococcus aureus* (MRSA) (n = 36), *Enterobacter species* (*spp*.) (n = 35), *Proteus mirabilis* (*P. mirabilis*) (n = 28), *C. freundii* (n = 23), *Pseudomonas aeruginosa* (*P. aeruginosa*) (n = 16), and vancomycin-resistant *enterococcus* (VRE) (n = 16). All *E. coli* and *K. pneumoniae* isolates were ESBL producers, and some of the *K. pneumoniae* isolates were also carbapenem-resistant. The isolates were identified to the species level by conventional biochemical testing and matrix-assisted laser desorption/ionization time-of-flight.

Extended spectrum beta-lactamase production was confirmed by a double disk synergy test according to Jarlier (7). Deformation of the inhibition zones around cephalosporin disks toward central disk containing clavulanic acid represented a positive result. *In vitro* sensitivity of multiresistant bacterial strains to fosfomycin was tested according to Clinical and Laboratory Standards Institute methodology (8). Thus, minimum inhibitory concentration (MIC) susceptibility breakpoint for fosfomycin was defined as 64 mg/L. Intermediate susceptibility was defined as MIC 128 mg/L and resistance as MIC≥256 mg/L. Fosfomycin MIC breakpoints were determined by agar dilution method, as this is the reference method for *in vitro* sensitivity testing to fosfomycin. Antimicrobial susceptibility testing to other routinely used antibiotics for UTI was performed by disk diffusion method using commercially-prepared, fixed concentration, paper antibiotic disks, in accordance with Clinical and Laboratory Standards Institute criteria (8).

Extended-spectrum beta-lactamases in *K. pneumoniae* were characterized by polymerase chain reaction and sequencing of beta-lactamase genes as described previously (9-12). Carbapenemase-producing *Enterobacteriaceae* were characterized in the previous studies (13-15).

We determined our sample size based on similar *in vitro* susceptibility studies (16-18). Statistical processing was carried out using the statistical package IBM SPSS Statistics, version 23 (IBM, Armonk, NY, USA, license owned by the Croatian Institute of Public Health). χ^2^ test was used to determine whether there was a significant difference between the susceptibility rates of multiresistant bacterial isolates to fosfomycin and other antibiotics. The level of significance was set at *P* < 0.05.

## Results

Susceptibility to fosfomycin, along with susceptibility to other antibiotics, was tested in 288 multiresistant bacterial isolates (236 Gram-negative and 52 Gram-positive). Out of 288 isolates, 238 (82.6%) were susceptible to fosfomycin. High susceptibility rates were found in both Gram-negative (199/236, 84.3%) and Gram-positive (39/52, 75%) isolates.

All of the 72 *E. coli* isolates were ESBL producers. Of those, 70 (97.2%) were susceptible to fosfomycin, with the remaining two intermediately susceptible (Table 1). No resistance to carbapenems was observed. The difference in susceptibility rates between fosfomycin and all other antibiotics, except carbapenems, was significant (χ^2^ test, *P* = 0.001).

**Table 1:** *In vitro* susceptibility rates to different antibiotics in different species of *Enterobacteriaceae* (n = 224)*

	Extended spectrum β-lactamase producing *Escherichia coli* (n = 72)			Extended spectrum β-lactamase producing *Klebsiella pneumoniae* (n = 50)			*Enterobacter cloacae* (n = 35)			*Proteus mirabilis* (n = 28)			*Citrobacter freundii* (n = 23)			*Pseudomonas aeruginosa* (n = 16)		
**Antibiotic**	**R**	**I**	**S**	**R**	**I**	**S**	**R**	**I**	**S**	**R**	**I**	**S**	**R**	**I**	**S**	**R**	**I**	**S**
Amoxicillin	72	0	0	50	0	0	35	0	0	28	0	0	23	0	0	NT	NT	NT
Amoxicillin/clavulanic acid	33	6	33	36	7	7	34	0	1	26	0	2	23	0	0	NT	NT	NT
Piperacillin/tazobactam	15	15	42	29	12	9	28	4	3	0	10	18	21	2	0	13	0	3
Cefazolin	72	0	0	50	0	0	35	0	0	28	0	0	23	0	0	NT	NT	NT
Cefuroxime	72	0	0	50	0	0	35	0	0	28	0	0	23	0	0	NT	NT	NT
Ceftazidime	61	5	6	50	0	0	34	0	1	26	1	1	23	0	0	13	0	3
Ceftriaxone	70	0	2	50	0	0	35	0	0	27	1	0	22	1	0	NT	NT	NT
Cefotaxime	NT	NT	NT	50	0	0	35	0	0	NT	NT	NT	23	0	0	NT	NT	NT
Cefoxitin	NT	NT	NT	NT	NT	NT	35	0	0	NT	NT	NT	NT	NT	NT	NT	NT	NT
Cefepime	43	11	18	23	14	13	24	8	3	1	2	25	19	3	1	11	0	5
Imipenem/cilastatin	0	0	72	0	0	50	26	2	7	0	0	28	14	4	5	13	2	1
Meropenem	0	0	72	0	0	50	28	0	7	0	0	28	14	4	5	15	1	0
Ertapenem	1	1	70	3	0	47	28	0	7	NT	NT	NT	17	1	5	NT	NT	NT
Gentamicin	30	0	42	44	0	6	32	1	2	26	1	1	16	2	5	15	1	0
Amikacin	NT	NT	NT	NT	NT	NT	10	8	17	NT	NT	NT	3	4	16	NT	NT	NT
Ciprofloxacin	64	0	8	41	1	8	32	3	0	27	1	0	16	0	7	14	2	0
Norfloxacin	64	0	8	42	0	8	NT	NT	NT	NT	NT	NT	NT	NT	NT	NT	NT	NT
Cotrimoxazole	61	0	11	31	3	16	NT	NT	NT	NT	NT	NT	NT	NT	NT	NT	NT	NT
Nitrofurantoin	22	1	49	NT	NT	NT	NT	NT	NT	NT	NT	NT	NT	NT	NT	NT	NT	NT
Colistin	NT	NT	NT	NT	NT	NT	0	0	35	NT	NT	NT	0	0	23	0	0	16
Fosfomycin	0	2	70	6	4	40	3	2	30	1	1	26	0	1	22	7	3	6

*R – resistant; I – intermediately susceptible; S – sensitive; NT – not tested.

The majority of 50 *K. pneumoniae* isolates were susceptible (40/50; 80%) or intermediately susceptible (4/50; 8%) to fosfomycin (Table 1). When compared to *E. coli* ESBL, *K. pneumoniae* isolates exhibited a much lower susceptibility to gentamicin (12% vs 58.3%), amoxicillin/clavulanic acid (14% vs 45.8%), and piperacillin/tazobactam (18% vs 58.3%). Susceptibility of *K. pneumoniae* to fosfomycin was also much lower, but still rather high, and the difference in susceptibility rates between fosfomycin and all other antibiotics, except carbapenems, was significant (χ^2^ test, *P* = 0.001). Six out of 50 (11.1%) ESBL-producing *K. pneumoniae* isolates were resistant to fosfomycin.

Among the *Enterobacter* *spp*. isolates, the susceptibility rate to fosfomycin was very high (30/35, 85.7%). Since the majority of isolates were carbapenem-resistant, they were susceptible only to colistin and fosfomycin (Table 1). Fosfomycin showed significantly better rates of *in vitro* activity than amikacin and all other antibiotics except colistin (χ^2^ test, *P* = 0.001). Resistance to amoxicillin/clavulanic acid, cefazolin, and cefuroxime observed in all isolates is due to the production of intrinsic, chromosomal AmpC beta-lactamase of *Enterobacter* *spp*.

Of the 23 extensively resistant *C. freundii* isolates, all were susceptible to colistin and all but one to fosfomycin (Table 1). The difference in susceptibility rate between fosfomycin (22/23) and amikacin (16/23) was significant (χ^2^ test, *P* = 0.021).

The great majority of multiresistant *P. mirabilis* isolates was susceptible to fosfomycin (26/28), carbapenems (28/28), and cefepime (25/28) (Table 1). This is because of the production of plasmid mediated-AmpC beta-lactamase, which hydrolyzes all beta lactams except fourth generation cephalosporins and carbapenems. The strains resistant to the third-generation cephalosporins were positive for CMY-16 (19).

More than 70% of carbapenem-resistant isolates demonstrated resistance against all commonly used antibiotics except fosfomycin (17.8%) and colistin (0%) (Table 2). The prevalence of fosfomycin resistance was significantly lower than the prevalence of resistance to all other antibiotics except colistin (χ^2^ test, *P* < 0.001). Resistance to colistin was significantly lower than resistance to fosfomycin (χ^2^ test, *P* < 0.001).

**Table 2:** *In vitro* susceptibility rates to different antibiotics in carbapenem-resistant *Enterobacteriaceae* and *Pseudomonas aeruginosa* (n = 73)*

	*Enterobacter cloacae* (n = 28)			*Citrobacter freundii* (n = 17)			*Pseudomonas aeruginosa* (n = 16)			*Klebsiella pneumoniae* (n = 12)			All isolates (%)
Antibiotic	R	I	S	R	I	S	R	I	S	R	I	S	R
Amoxicillin	28	0	0	17	0	0	NT	NT	NT	12	0	0	57/57 (100)
Amoxicillin/clavulanic acid	28	0	0	17	0	0	NT	NT	NT	12	0	0	57/57 (100)
Piperacillin/tazobactam	26	2	0	17	0	0	13	0	3	12	0	0	68/73 (93.1)
Cefazolin	28	0	0	17	0	0	NT	NT	NT	12	0	0	57/57 (100)
Cefuroxime	28	0	0	17	0	0	NT	NT	NT	12	0	0	57/57 (100)
Ceftazidime	28	0	0	17	0	0	13	0	3	12	0	0	70/73 (95.9)
Ceftriaxone	28	0	0	17	0	0	NT	NT	NT	12	0	0	57/57 (100)
Cefotaxime	28	0	0	17	0	0	NT	NT	NT	12	0	0	57/57 (100)
Cefepime	22	6	0	15	2	0	11	0	5	12	0	0	60/73 (82.2)
Imipenem/cilastatin	26	2	0	13	4	0	13	2	1	9	1	2	61/73 (83.6)
Meropenem	28	0	0	13	4	0	15	1	0	11	0	1	67/73 (91.8)
Ertapenem	28	0	0	16	1	0	NT	NT	NT	12	0	0	56/57 (98.2)
Gentamicin	25	1	2	11	2	4	15	1	0	4	0	7	56/73 (76.7)
Ciprofloxacin	27	1	0	12	0	5	14	2	0	11	0	1	64/73 (87.7)
Colistin	0	0	28	0	0	17	0	0	16	0	0	12	0/73 (0)
Fosfomycin	2	1	25	0	1	16	7	3	6	4	3	5	13/73 (17.8)

*R – resistant; I – intermediately susceptible; S – sensitive; NT – not tested.

Methicillin-resistant *Staphylococcus aureus* isolates were susceptible to linezolide (36/36), tigecycline (36/36), vancomycin (36/36), teicoplanine (36/36), trimethoprim-sulfamethoxazole (35/36), fosfomycin (34/36), and rifampicin (34/36). Only two of 36 isolates showed intermediate susceptibility to fosfomycin, while all other were sensitive (Table 3). The difference in the prevalence of resistance between fosfomycin (2/36) and all of the following antibiotics was significant: ciprofloxacin (31/36), azithromycin (28/36), clarithromycin (28/36), clindamycin (25/36), and gentamicin (8/36) (χ^2^ test, *P* < 0.01 for all).

**Table 3:** *In vitro* susceptibility rates to different antibiotics in methicillin-resistant *Staphylococcus aureus* (n = 36)

	No. of isolates		
Antibiotic	resistant	intermediate	susceptible
Penicillin	36	0	0
Oxacillin	36	0	0
Gentamicin	8	0	28
Ciprofloxacin	31	0	5
Azithromycin	28	0	8
Clarithromycin	28	0	8
Clindamycin	25	0	11
Trimethoprim-sulfamethoxazole	1	0	35
Vancomycin	0	0	36
Teicoplanine	0	0	36
Rifampicin	2	0	34
Linezolide	0	0	36
Tigecycline	0	0	36
Fosfomycin	0	2	34

Only 5 of 16 VRE isolates tested demonstrated susceptibility to fosfomycin and all of these isolates had MIC 64 mg/L (Table 4).

**Table 4:** *In vitro* susceptibility rates to different antibiotics in vancomycin resistant *Enterococcus* (n = 16)

	No. of isolates		
Antibiotic	resistant	intermediate	susceptible
Ampicillin	16	0	0
Vancomycin	16	0	0
Teicoplanine	16	0	0
Linezolide	0	0	16
Fosfomycin	5	6	5

Multiresistant *P. mirabilis*, *C. freundii,* and ESBL producing *E. coli* isolates had low, and multiresistant *P. aeruginosa* and VRE isolates had high MIC_50_ and MIC_90_ of fosfomycin (Table 5). Rather unexpected findings were high MIC_50_ and MIC_90_ of fosfomycin in carbapenem-resistant *K. pneumoniae* isolates (128 and 512 mg/L, respectively). Extended spectrum β-lactamase-producing *K. pneumoniae* had significantly higher susceptibility to fosfomycin than carbapenem-resistant *K. pneumoniae* (χ^2^ test, *P* = 0.008).

**Table 5:** Minimum inhibitory concentration (MIC)_50_, MIC_90_, and *in vitro* susceptibility rates to fosfomycin in different multiresistant bacterial species

	MIC_50_ (mg/L)	MIC_90_ (mg/L)	No. (%) of susceptible isolates
*Proteus mirabilis*	2	16	26/28 (93)
*Citrobacter freundii*	4	16	22/23 (96)
Extended spectrum β-lactamase producing *Escherichia coli*	4	32	70/72 (97)
Methicillin-resistant *Staphylococcus aureus*	8	32	34/36 (94)
*Enterobacter species*	16	128	30/35 (86)
Extended spectrum β-lactamase producing *Klebsiella pneumoniae*	32	256	40/50 (80)
*Pseudomonas aeruginosa*	128	256	6/16 (38)
Vancomycin resistant *Enterococcus*	128	256	5/16 (31)
Carbapenem-resistant *Klebsiella pneumoniae*	128	512	5/12(42)

Eighty four percent of multiresistant Gram-negative isolates were *in vitro* susceptible to fosfomycin and 69% were susceptible to carbapenems. Susceptibility rates to other antibiotic groups were much lower (Table 6). The difference between susceptibility rates to fosfomycin and carbapenems was significant (χ^2^ test, *P* = 0.001).

**Table 6:** *In vitro* susceptibility rates for different antibiotics in Gram-negative multiresistant isolates (n = 236)

Antibiotic	No. of susceptible isolates (%)
Amoxicillin*	0/220 (0)
Amoxicillin/clavulanic acid*	43/220 (19.5)
Piperacillin/tazobactam	75/236 (31.8)
Cefazolin*	0/220 (0)
Cefuroxime*	0/220 (0)
Ceftazidime	11/236 (4.7)
Ceftriaxone*	0/220 (0)
Cefepime*	65/236 (27.5)
Imipenem/cilastatin	165/236 (69.9)
Meropenem	163/236 (69.1)
Ertapenem†	129/192 (67.2)
Gentamicin	63/236 (26.7)
Ciprofloxacin	24/236 (10.2)
Fosfomycin	199/236 (84.3)

*Pseudomonas aeruginosa isolates were not tested.

†*Pseudomonas aeruginosa* and *Proteus mirabilis* isolates were not tested.

We performed a polymerase chain reaction analysis of 36/50 ESBL producing *K. pneumoniae* isolates. The majority of isolates had CTX-M-15 beta lactamases and IS*Ecp* insertion (27/36) sequence, which enhances gene expression and level of resistance, and is important for the gene mobilization (20). All of the carbapenem-resistant *Enterobacter* and *Citrobacter* isolates were carrying *bla*_VIM-1_ metallo-beta-lactamase gene, while *Citrobacter* isolates also had chromosomaly-encoded CMY AmpC-type beta-lactamase. Of the 8 carbapenem-resistant *K. pneumoniae* isolates, one was oxacillinase-48 positive and the other had Verona integron-encoded metallo-β-lactamase-1 (Table 7).

**Table 7:** Beta-lactamase content of various multiresistant clinical isolates according to bacterial species*

ESBL-producing *K. pneumoniae* (n = 36)	No. of isolates	Carbapenem-resistant *Enterobacter species* (n = 28)	No. of isolates	Carbapenem-resistant *Citrobacter freundii* (n = 17)	No. of isolates	Carbapenem-resistant *Klebsiella pneumoniae* (n = 8)	No. of isolates
SHV-11, IS26CTX-M-3	1	VIM-1, TEM-1, CTX-M-15	18	VIM-1, TEM-1, CMY-4	13	VIM-1, SHV-1, CTX-M-15	1
SHV-11, IS*Ecp*CTX-M-15	1	VIM-1, TEM-1, SHV-1, CTX-M-15	1	VIM-1, TEM-1, CMY-2	1	VIM-1, SHV-1, TEM-1, CTX-M-15	2
IS*Ecp*CTX-M-15, TEM-1	1	VIM-1, TEM-1	3	VIM-1, TEM-1, CMY-4, CTX-M-15	3	VIM-1, CTX-M-15	1
SHV-1	3	VIM-1, NDM-1, TEM-1	1			VIM-1, TEM-1	1
SHV-1, TEM-1	3	VIM-1, NDM-1, TEM-1, CTX-M-15	1			VIM-1, NDM-1, SHV-1, TEM-1	3
SHV-1, IS26CTX-M-3	1	VIM-1, OXA-48, TEM-1, CTX-M-15	1			OXA-48, CTX-M-15, TEM-1	1
SHV-1, IS*Ecp*CTX-M-15	17	VIM-1, DHA	1				
SHV-1, IS*Ecp*CTX-M-15, TEM-1	6	VIM-1, CMY, TEM-1, CTX-M-15	1				
SHV-11, TEM-1	1	VIM-1, TEM-1, CMY-4	1				
SHV-11, IS*Ecp*CTX-M-15, TEM-1	2						

*ESBL – extended spectrum β-lactamase; CMY – cephamycinase; CTX-M – cefotaximase-Munich; DHA – a type of AmpC beta-lactamase; IS – insertion sequence; NDM – New Delhi metallo-beta-lactamase; OXA – oxacillinase; SHV – sulfhydryl variable; ST – sequence type; TEM – Temoniera; VIM – Verona integron-encoded metallo-β-lactamase.

## Discussion

The main finding of our study is high susceptibility rate to fosfomycin (82.6%) among 288 multiresistant isolates, which affirmed our hypothesis. Both the Gram-positive and Gram-negative isolates showed high rates of sensitivity to fosfomycin. An important finding of our study is that fosfomycin had significantly better *in vitro* activity against multiresistant Gram-negative isolates than carbapenems. Since Gram-negative pathogens are the most common causative agents of nosocomial bacterial infections, the results of our study have implications for empirical treatment of suspected bacterial infections in the hospital setting. This is in accordance with recent trends of the revival of old antibiotics such as colistin, fosfomycin, and nitrofurantoin, as these drugs still exhibit high *in vitro* activity against evolving multiresistant Gram-negative pathogens (21,22). While nitrofurantoin is only used clinically for the treatment of UTIs, both fosfomycin and colistin are also used in the treatment of other infections, with the advantages of fosfomycin being the additional coverage of Gram-positive pathogens and lack of nephrotoxicity (4). The more common use of fosfomycin and colistin in appropriate clinical setting represents one of the carbapenem-sparing strategies and is promoted by the leading experts in the field (23).

Both ESBL-producing *E. coli* and *K. pneumoniae* exhibited high susceptibility rates to fosfomycin, with the main difference being higher MICs in *K. pneumoniae* isolates. Among ESBL-producing *E. coli* isolates, fosfomycin demonstrated similar *in vitro* activity as carbapenems, which represent the first-line group of antibiotics for the treatment of infections caused by these pathogens (23). An acceptable alternative was nitrofurantoin, which, however, has more limited antibacterial spectrum than fosfomycin and carbapenems. Based on our results, the use of other antibiotics could not be routinely recommended. It needs to be emphasized that the two isolates with intermediate susceptibility to fosfomycin were obtained from patients previously treated with prolonged courses of fosfomycin owing to complicated UTIs. The emergence of fosfomycin resistance during prolonged treatment courses has been reported in the literature and is quoted as one of the main concerns regarding the clinical utility of fosfomycin (24). The susceptibility rate of ESBL-producing isolates to quinolones and trimethoprim-sulfamethoxazole was especially low, as expected since plasmids encoding ESBLs usually carry genes responsible for resistance to quinolones, sulphonamides, and aminoglycosides. The percentage of ESBL production is considerable, not only in hospitalized patients but also in outpatients. Based on the surveillance data from the Reference Centre for Antibiotic Resistance Surveillance of the Croatian Ministry of Health, 47% of *K. pneumoniae* and 13% of *E. coli* invasive isolates were ESBL producers in 2015, with a rise of ESBL production in *E. coli* compared to earlier years (25). Fosfomycin has been widely accepted as the first-line antibiotic treatment of uncomplicated UTIs (1). The current Croatian national guidelines also recommend the use of fosfomycin as one of the treatment options for acute uncomplicated lower UTIs in women and for UTIs in pregnant women (26). Since the resistance to trimethoprim-sulfamethoxazole is high in *E. coli* and *K. pneumoniae* with and without ESBL production, fosfomycin and nitrofurantoin could be taken into the consideration as the first-line therapy for the UTIs caused by multiresistant pathogens.

Falagas et al (4) systematically reviewed 17 studies evaluating the antimicrobial activity of fosfomycin. Using a MIC susceptibility breakpoint of 64 mg/L, they found 1604 (96.8%) of 1657 ESBL-producing *E. coli* isolates and 608 (81.3%) of 748 ESBL-producing *K. pneumoniae* isolates to be susceptible to fosfomycin. We obtained similar results, with 97.2% and 80% of ESBL-producing *E.coli* and *K. pneumoniae* being susceptible to fosfomycin, respectively.

An unexpected finding is a high resistance to fosfomycin in carbapenem-resistant *K. pneumoniae* isolates (MIC_50_ and MIC_90_ 128 and 512 mg/L, respectively). Although only 12 isolates were tested, MICs were higher than in VRE or *P. aeruginosa* isolates, which are expected to be more resistant. These isolates also had significantly lower susceptibility rate than ESBL-producing *K. pneumoniae* isolates. Since fosfomycin has a unique mechanism of action, it does not display cross-resistance with other antibiotics. Hence, this finding is not in concordance with the literature and should be confirmed on a larger number of isolates (27).

Our results show excellent fosfomycin activity against *C. freundii* isolates and are similar to those by Samonis et al (28), who found all 29 *C. freundii* isolates to be susceptible to fosfomycin. However, in the latter study, the majority of isolates were susceptible to cephalosporins and fluoroquinolones, and all but one isolate were susceptible to imipenem, while most of our isolates were resistant to carbapenems (28). Our results are similar to those by Hammerum et al (29), who tested 13 New Delhi metallo-beta-lactamase 1-producing *C. freundii* isolates, all of which were susceptible to fosfomycin.

Fifty-two of 73 isolates (71.2%) of carbapenem-resistant *Enterobacteriaceae* were susceptible to fosfomycin. The rate of susceptibility was higher than that reported by Livermore et al (30), who found 49/81 isolates (60.5%) to be *in vitro* sensitive to fosfomycin. The difference could be explained by the fact that their sample included more *Klebsiella spp*. isolates (64.2% of the whole sample), while ours included more *Enterobacter* *spp.* and *C. freundii* isolates, with lower MICs for fosfomycin (*Enterobacter* *spp*. and *C. freundii* accounted for 62% of all carbapenem-resistant isolates).

Fosfomycin activity against *Enterobacter spp.* was comparable to the results by Kaase et al (5). *Pseudomonas* isolates are known to have high MICs of fosfomycin. Fosfomycin had similar activity as amikacin and better activity than carbapenems, but the number of tested isolates was small.

Among Gram-positive isolates, we found excellent susceptibility rates for MRSA and low for VRE, which is in accordance with the data published earlier (3).

The main limitation of our study is the fact that, owing to financial restrictions, not all isolates were analyzed by pulsed-field gel electrophoresis. Hence, it cannot be excluded that some of the isolates were genetically identical and hence represent a clone. Moreover, the susceptibility of some isolates was not tested to all antibiotics. For example, susceptibility to amikacin was not tested in *P. mirabilis*, *P. aeruginosa,* and ESBL-producing *E. coli* and *K. pneumoniae* isolates.

According to our results, fosfomycin is a potentially effective treatment option for infections caused by multiresistant Gram-negative and Gram-positive bacterial isolates, with the exception of VRE. Fosfomycin demonstrated the best *in vitro* activity of all antibiotics tested against 288 multiresistant bacterial isolates. Hence, it is a valuable addition to antibiotic armamentarium in the hospital setting, especially for Gram-negative infections, in which antimicrobial resistance rates are rising and effective antibiotic options are scarce. In the context of the worrisome high rates of carbapenem-resistance among multiresistant Gram-negative isolates, fosfomycin represents a potentially valuable treatment option, both clinically and as a carbapenem-sparing strategy.
